# Post-Distillation By-Products of Aromatic Plants from Lamiaceae Family as Rich Sources of Antioxidants and Enzyme Inhibitors

**DOI:** 10.3390/antiox12010210

**Published:** 2023-01-16

**Authors:** Simon Vlad Luca, Gokhan Zengin, Kouadio Ibrahime Sinan, Krystyna Skalicka-Woźniak, Adriana Trifan

**Affiliations:** 1 Biothermodynamics, TUM School of Life Sciences, Technical University of Munich, 85354 Freising, Germany; 2 Physiology and Biochemistry Research Laboratory, Department of Biology, Science Faculty, Selcuk University, Konya 42130, Turkey; 3 Department of Natural Products Chemistry, Medical University of Lublin, 20-093 Lublin, Poland; 4 Department of Pharmacognosy and Phytotherapy, Faculty of Pharmacy, “Grigore T. Popa” University of Medicine and Pharmacy Iasi, 700115 Iasi, Romania

**Keywords:** *Ocimum basilicum* L., *Origanum vulgare* L., *Thymus vulgaris* L., antioxidant, anti-enzymatic, by-products, waste, LC-HRMS/MS, GC-MS

## Abstract

There is currently no use for the vast quantities of post-distillation by-products, such as spent plant materials and residual waters, produced by the essential oil (EO) industry of aromatic herbs. In this study, the EOs of three Lamiaceae species (thyme, oregano, and basil) and their total, spent, and residual water extracts were phytochemically characterized and biologically assessed. The collected information was put through a series of analyses, including principal component analysis, heatmap analysis, and Pearson correlation analysis. Concerning the EOs, 58 volatile compounds were present in thyme (e.g., *p*-cymene, thymol), 44 compounds in oregano (e.g., thymol, carvacrol), and 67 compounds in basil (e.g., eucalyptol, linalool, estragole, (*E*)-methyl cinnamate). The LC-HRMS/MS analysis of the total, spent, and residual water extracts showed the presence of 31 compounds in thyme (e.g., quercetin-*O*-hexoside, pebrellin, eriodictyol), 31 compounds in oregano (e.g., rosmarinic acid, apigenin, kaempferol, salvianolic acids I, B, and E), and 25 compounds in basil (e.g., fertaric acid, cichoric acid, caftaric acid, salvianolic acid A). The EOs of the three Lamiaceae species showed the highest metal-reducing properties (up to 1792.32 mg TE/g in the CUPRAC assay), whereas the spent extracts of oregano and basil displayed very high radical-scavenging properties (up to 266.59 mg TE/g in DPPH assay). All extracts exhibited anti-acetylcholinesterase (up to 3.29 mg GALAE/g), anti-tyrosinase (up to 70.00 mg KAE/g), anti-amylase (up to 0.66 mmol ACAE/g), and anti-glucosidase (up to 1.22 mmol ACAE/g) effects. Thus, the present research demonstrated that both the raw extracts (EOs and total extracts) and the post-distillation by-products (spent material and residual water extracts) are rich in bioactive metabolites with antioxidant and enzyme inhibitory properties.

## 1. Introduction

Aromatic plants/herbs are important constituents of human nutrition, valued as agents of aroma, flavor, color, and preservation in food. Additionally, aromatic plants contain various phytochemicals endowed with potent biological activity, making them promising candidates for the treatment of a variety of chronic diseases [1]. The culinary, cosmeceutical, and medicinal uses of plants in the Lamiaceae family have made them stand out among aromatic herbs over the years [2].

*Thymus* L. is one of the largest genera of the Lamiaceae family, with over 350 species found in Europe, Asia, and North Africa [3]. Among them, the most significant species is (common) thyme (*T. vulgaris* L.), which is cultivated worldwide for its culinary, cosmetic (additive in deodorants, toothpaste, and mouthwash), and medicinal uses. Ethnomedicinally, thyme leaves are used to prevent and treat gastrointestinal and respiratory problems, such as colds, flu, indigestion, nausea, and dysentery [4]. Thyme is reported to contain more than 90 volatile compounds (e.g., monoterpenes, sesquiterpenes, alcohols, aldehydes, ketones, esters); of these, thymol and carvacrol constitute ~75% of its total volatile fraction [5]. In addition to the volatile compounds usually isolated in the form of essential oil (EO), studies have also indicated the presence of phenolic compounds, in particular, phenolic acids (e.g., caffeic acid, ferulic acid, danshensu, etc.) and flavonoids (luteolin, apigenin, cirsilineol, salvigenin, and their glycosides, etc.) [3,4,6]. These compounds are responsible for the antioxidant, antimicrobial, anti-inflammatory, spasmolytic, neuroprotective, cardio-vasculo-protective, or anticancer properties of various thyme leaf extracts [7,8,9].

*Origanum* L. (Lamiaceae) is a genus of about 43 plant species that are native to various parts of the world, including the Mediterranean, tropical and temperate Asia, Africa, and naturalized populations in India, North America, and Mexico. [10,11]. The most common species (*O. vulgare* L., oregano) plays a significant role among aromatic herbs due to its characteristic odor and flavor of the flowers and leaves [12]. In folk medicine, oregano is also used to treat gastrointestinal disorders (e.g., indigestion, abdominal pain), respiratory disorders (e.g., colds, bronchial disorders), infections, etc. [13]. Phytochemical studies have evidenced the presence of volatile compounds in the EO (e.g., monoterpenes, sesquiterpenes), diterpenoids, triterpenoids, phenolic acids (e.g., chlorogenic acid, coumaroylquinic acid, feruloylquinic acid, syringic acid, etc.), and flavonoids (e.g., quercetin, apigenin, luteolin, gallocatechin, eriodictyol, taxifolin, and their glycosides) [14,15,16]. These constituents are assumed to be responsible for the observed antioxidant, antimicrobial, anti-inflammatory, anticancer, neuroprotective, anti-urolithic, anti-hyperlipidemic, and anti-nociceptive properties of various *Origanum* species [11,14,17,18].

More than 64 plant species native to Asia, Africa, and Central and South America are included in the *Ocimum* L. genus (Lamiaceae) [19]. The EO of basil (*O. basilicum* L.) has extensive applications in the culinary, cosmetics, pharmaceutical, flavor, fragrance, perfume, and toiletry industries [20]. In traditional medicine, basil preparations are administered to treat cough, headache, diarrhea, and skin infections [21]. In addition to the terpenes present in the EO (e.g., eugenol, eucalyptol, fenchone, estragole, etc.), flavonoids (e.g., quercetin, luteolin, orientin, cirsiliol, cirsilineol, kaempferol, apigenin, and their glycosides) and phenolic compounds (e.g., caffeic acid, ferulic acid, sinapic acid, caftaric acid, rosmarinic acid, etc.) prevail in basil aerial part extracts [19,22,23,24,25]. Furthermore, biological studies on basil revealed a diverse pharmacological profile, including antimicrobial, gastroprotective, anticancer, antidiabetic, and anti-hyperlipidemic properties. [20,26,27,28].

The extraction of EOs constitutes one of the main reasons for the large-scale cultivation of thyme, oregano, and basil. Due to their low cost, simplicity, and generation of high-quality oils, steam distillation and hydrodistillation continue to be the primary technologies that satisfy industrial needs for EOs [2]. However, various post-distillation by-products, including spent plant materials (solid residues, waste biomass), aqueous condensates (hydrolates, hydrosols), and distillation wastewaters (residual waters or leachates), are generated in large amounts. Usually, these by-products are considered wastes and discarded in the environment without further processing [29]. Therefore, there can be substantial economic and ecological effects from finding ways to utilize the by-products left after distillation. Recent research has shown that the by-products of distillation can be a valuable low-cost source of high-added-value products. [1,8,29,30,31,32,33]. The extraction of pharmacologically active molecules, such as phenolic compounds, provides a novel method of valorizing such waste materials [34].

Literature data on the recovery of post-distillation by-products from thyme, oregano, and basil are quite scarce. Only a few reports documented the development of modern analytical tools to identify and quantify phenolic compounds extracted from the spent materials of oregano [34]. The metabolite profiling and evaluation of the antioxidant and antimicrobial activity of decocts and macerates from the spent materials of thyme and basil were also performed [2,8,35].

However, to the best of the authors’ knowledge, a comparative assessment of the phytochemical profile and biological potential (e.g., antioxidant, enzyme inhibition) of the EOs, total, and post-distillation extracts from thyme, oregano, and basil is lacking. Thus, in the current study, the EOs from the above-mentioned Lamiaceae aromatic herbs were characterized by gas chromatography coupled with mass spectrometry (GC-MS), whereas the total, spent, and residual water extracts were profiled by liquid chromatography coupled with high-resolution tandem mass spectrometry (LC-HRMS/MS). To evaluate the in vitro biological potential, six antioxidant tests [1,1′-diphenyl-2-picrylhydrazyl (DPPH), 2,2′-azino-bis(3-ethylbenzothiazoline) 6-sulfonic acid (ABTS), cupric ion reducing antioxidant capacity (CUPRAC), ferric ion reducing antioxidant power (FRAP), metal chelating ability (MCA), phosphomolybdenum (PBD)] and five enzyme inhibition tests [acetylcholinesterase (AChE), butyrylcholinesterase (BChE), tyrosinase, amylase, glucosidase] were carried out.

## 2. Materials and Methods

### 2.1. Plant Material

Dried aerial parts of thyme (*Thymus vulgaris* L.), oregano (*Origanum vulgare* L.), and basil (*Ocimum basilicum* L.) were purchased from local markets in Germany; their identity was confirmed by one of the authors (A.T.). Vouchers (TV/220714, OV/220714, OB/220714) were stored in the Department of Pharmacognosy and Phytotherapy, Faculty of Pharmacy, “Grigore T. Popa” University of Medicine and Pharmacy Iasi, Romania.

### 2.2. Extraction

#### 2.2.1. Essential Oil Isolation

The powdered plant material (50 g from each species) was subjected to hydrodistillation with 500 mL of deionized water for 4 h in a Clevenger-type apparatus. The obtained essential oils (EOs) were measured (with the scale of the apparatus in mL), collected, dried over anhydrous sodium sulfate, and stored in dark glass tubes at 4 °C until further analysis. Each procedure was performed in duplicate.

#### 2.2.2. Preparation of Total, Spent, and Residual Water Extracts

At the end of the hydrodistillation process, the water in the flask was filtered, and aliquots (25 mL) were freeze-dried, yielding the residual water extracts (WE). The residue (spent plant material) was dried in an oven at 40 °C for 48 h, and 10 g was extracted with 3 × 100 mL of methanol/water 75/25 (*v*/*v*) by ultra-sonication (30 min). After filtration, the obtained extracts (spent extracts, SE) were evaporated to dryness under vacuum and kept at −20 °C until further analysis. For comparison purposes, unprocessed powdered plant material (10 g from each species) was extracted similarly with 3 × 100 mL of methanol/water 75/25 (*v*/*v*) by ultra-sonication (30 min). After filtration, the obtained extracts (total extracts, TE) were evaporated to dryness under vacuum and kept at −20 °C until analysis. Each procedure was performed in duplicate.

The extraction yields to obtain the EOs, WE, SE, and TE from thyme, oregano, and basil are provided in Table 1.

### 2.3. GC-MS Analysis

The analyses were performed on a TRACE gas chromatograph (GC) coupled to an ISQ™ mass spectrometer (MS) from Thermo Fisher (Waltham, MA, USA). The column was Zebron™ ZB-5MS (30 m × 0.25 mm i.d., 0.25 µm film thickness) from Phenomenex (Torrance, CA, USA); Carrier gas—helium; flow rate—1.43 mL/min; inlet temperature—250 °C; split ratio—50:1; injection volume—1 μL. The oven temperature was programmed as follows: 4 min held at 60 °C; then ramped up to 280 °C at a rate of 10 °C/min and held at 280 °C for 5 min; then ramped to 300 °C at 10 °C/min and held at 300 °C for 10 min. The MS was set in full scan mode from 50–350 amu, with the following parameters: ionization energy—70 eV; source temperature—230 °C; transfer line temperature—320 °C. Linear retention indices (LRI) were calculated for the individual compounds using a standard mixture of C8–C20 *n*-alkanes. The compounds were identified by comparing their mass spectra with those from the NIST 11 Mass Spectra Library and their LRI with literature data. Each analysis was performed in triplicate.

### 2.4. LC-HRMS/MS Analysis

The analyses were performed on an Agilent 1200 HPLC (Agilent Technologies, Palo Alto, CA, USA) comprising a binary pump (G1312C), column thermostat (G1316A), auto-sampler (G1329B), and accurate-mass quadrupole-time-of-flight MS detector (G6530B). The column was a Gemini C18 (100 mm × 2 mm i.d., 3 μm) from Phenomenex (Torrance, CA, USA). A linear gradient composed of 0.1% formic acid in water (A) and 0.1% formic acid in acetonitrile (B) was applied as follows: 10% B (0 min), 60% B (45 min), 95% B (46–55 min); flow rate—0.2 mL/min; column temperature—20 °C; injection volume—10 μL. The MS was set in full scan mode from 100–1700 amu, with the following parameters: negative ionization mode; gas (nitrogen) flow rate—10 L/min, gas temperature—275 °C; sheath gas (nitrogen) flow rate—12 L/min; sheath gas temperature—325 °C; nebulizer pressure—35 psi; capillary voltage—4000 V; nozzle voltage—1000 V; skimmer—65 V; fragmentor—140 V; collision-induced dissociation—10 and 30 V. The assignment of the peaks observed in the base peak chromatograms (BPC) was performed by comparing the spectrometric data with previous literature data reporting on the LC-MS analysis of similar constituents or online databases (METLIN, KNApSacK, PubChem, NIST Chemistry WebBook).

### 2.5. Total Phenolic, Flavonoid, Antioxidant, and Enzyme Inhibition Assays

Total phenolic content (TPC), total flavonoid content (TFC), DPPH radical scavenging, ABTS radical scavenging, cupric reducing antioxidant capacity (CUPRAC), ferric reducing antioxidant power (FRAP), metal chelating activity (MCA), phosphomolybdenum (PBD), inhibition of acetylcholinesterase (AChE), butyrylcholinesterase (BChE), tyrosinase, amylase, and glucosidase assays were performed as previously described [36,37]. Each sample was processed in triplicate.

### 2.6. Statistical and Data Processing

Data are presented as mean ± standard deviation of the number of replicates. One-way analysis of variance with Tukey’s post-hoc test was conducted; *p* < 0.05 was considered statistically significant. The relationship between the molecules and antioxidant activities and enzyme inhibitory activities was assessed by calculating the Pearson correlation coefficient. Pearson coefficient values >0.8 were considered significant. Afterward, the principal component analysis (PCA) and Heatmap analysis were performed. Before the PCA, the LC-HRMS/MS data (peak areas from the base peak chromatograms) were log-transformed. The statistical and data processing was done using R software v. 4.1.2.

## 3. Results and Discussion

### 3.1. GC-MS Characterization of Essential Oils

Aerial parts of thyme, oregano, and basil were subjected to hydrodistillation as described in Section 2.2.1. The highest extraction oil yield was noticed for oregano, followed by thyme and basil (Table 1). Subsequently, the EOs obtained from the three Lamiaceae species were analyzed by GC-MS (Table 2).

Fifty eight volatile compounds were identified in the thyme EO, accounting for ~99% of the total constituents (as assessed from the GC-MS chromatograms). Most of these were monoterpenes (93.74%), with considerably fewer sesquiterpenes (5.59%). Thus, the main constituents from the thyme EO were thymol (40.26%), *p*-cymene (25.70%), followed by γ-terpinene (4.89%), and carvacrol (4.48%). This is consistent with the chemical composition reported in previous studies. For instance, Micucci et al. [7] reported amounts of thymol, carvacrol, *p*-cymene, and γ-terpinene of 43.3%, 20.7%, 9.4%, and 4.0%, respectively. Similarly, Sonmezdag et al. [5] also found thymol (61.74–78.82%), *p*-cymene (8.92–17.90%), and carvacrol (4.41–5.62%) to be the representative volatile compounds in *T. vulgaris* EO.

In the case of oregano EO, 44 compounds accounted for up to 99% of the total peaks observed in the GC-MS chromatograms (Table 2). Similar to the thyme EO, 95.03% were monoterpenes, whereas 4.74% were sesquiterpenes. Carvacrol (69.93%) was the dominant constituent, followed by thymol (9.40%) and *p*-cymene (6.71%). These findings are consistent with previous reports. Ozdemir et al. [16] showed that *O. vulgare* EOs obtained from differently processed plant materials contained levels of carvacrol between 45.09–46.71% and thymol between 14.67–15.72%. Carvacrol (72.8–79.5%) was also shown as the main constituent in the EO obtained from the leaves and stems of oregano [17].

Basil EO was characterized by 67 compounds, accounting for ~99% of the peaks in the GC-MS chromatograms. Monoterpenes (54.32%) were also the predominant class of volatile compounds; however, as opposed to the thyme and oregano EOs that contained <5% sesquiterpenes, the sesquiterpenes in the basil EO were considerable, at 31.06%. In addition to three monoterpenes, estragole (19.62%), linalool (17.70%), and eucalyptol (5.46%), and two sesquiterpenes, (*Z*)-α-bergamotene (7.11%) and α-cadinol (5.64%), (*E*)-methyl cinnamate (12.06%), a phenylpropanoid compound was also found in significantly high amounts in the basil EO (Table 2). Methyl cinnamate (15.42–64.69%) and linalool (12.47–15.18%) were previously reported to be the major volatile constituents in the *O. basilicum* EO [25]. In addition, Hong et al. [26] evidenced the presence of linalool, eucalyptol, and β-santalene as the prevalent terpenes in the basil EO.

### 3.2. LC-HRMS/MS Analysis of Total, Spent, and Residual Water Extracts

The total (unspent plant material) extracts (TE), residual water extracts (WE), and spent plant material (solid residue) extracts (SE) were obtained from thyme, oregano, and basil, as described in Section 2.2.2. For all samples, the highest extraction yields were obtained for the WE, followed by the TE and SE (Table 1). All extracts were phytochemically profiled by LC-HRMS/MS, with the spectro-chromatographic data provided in Table 3.

A total of 31 specialized metabolites belonging to various phytochemical classes were ascribed to the thyme extracts. Danshensu (**2**), hydroxybenzoic acid (**4**) and its hexoside (**2**), caffeic acid (**9**) and its hexoside (**6**), and rosmarinic acid (**26**) were accounted as phenolic acids. Similar metabolites were already reported in various *Thymus* species extracts [5,8,38]. Flavonoids were the most abundant category of compounds, as follows: free aglycones: taxifolin (**20**), gallocatechin (**23**), eriodictyol (**32**), luteolin (**35**), cirsimaritin (**38**), pebrellin (**44**), and cirsilineol (**45**); *O*-glycosides: aromadendrin-*O*-hexoside (**15**), quercetin-*O*-hexosides (**18** and **22**); and *C*-glycosides: luteolin-*C*-deoxyhexoside-*C*-hexoside (**14**). Free aglycones, *O*- and *C*-glycosides were previously reported in the *Thymus* genus [3,5,38]. Two biphenyls, 4′-hydroxy-5,5′-diisopropyl-2,2′-dimethyl-3,4-biphenylquinone (**50**) and 3,4,4′-trihydroxy-5,5′-diisopropyl-2,2′-dimethylbiphenyl (**52**) were also found in the WE, SE, and TE of thyme. These two derivatives were previously isolated from *T. vulgaris* by Nakatani et al. [39]. Several terpenes were tentatively identified in the current thyme extracts, such as two monoterpene glycosides (*p*-menth-1-ene-3,4-diol-*O*-hexoside, **16**, and cymenol-*O*-hexoside, **30**) and two diterpenes (carnosol, **47** and dehydrocarnosol, **48**). Lastly, three fatty acid derivatives were putatively ascribed as follows: tuberonic acid (**11**), hydroperoxyoctadecadienoic acid (**46**), and hydroxyoctadecadienoic acid (**51**). The three thyme extracts (WE, TE, and SE) displayed a very similar qualitative profile. Hydroxybenzoic acid-*O*-hexoside (**3**) was absent in the TE, whereas aromandendrin-*O*-hexoside (**15**) and taxifolin (**20**) were not present in the WE and SE. One of the non-polar biphenyls (**50**) and the lipophilic fatty acid **51** were not observed in the WE (Table 3).

Oregano extracts were characterized by 31 specialized metabolites (Table 3). Similar to the thyme extracts, quinic acid (**1**), danshensu (**2**), hydroxybenzoic acid-*O*-hexoside (**3**), hydroxybenzoic acid (**4**), caffeic acid-*O*-hexoside (**6**), and rosmarinic acid (**26**) were also present in the oregano extracts. In addition, six salvianolic acids, namely salvianolic acid H (**21**), salvianolic acid D (**25**), salvianolic acid B (**27**), salvianolic acid A (**28**), salvianolic acid I (**31**), and salvianolic acid E (**33**) were tentatively annotated in the oregano extracts. Previously, phenolic acids, including salvianolic acids, were identified as common metabolites of *Origanum* species. [11,14,15]. The two biphenyls **50** and **52**, previously not reported in the *Origanum* genus, were also noticed. The seven flavonoids spotted in the oregano extracts can be sub-grouped into free aglycones: taxifolin (**20**), gallocatechin (**23**), apigenin (**37**), and kaempferol (**41**); *O*-glycosides: luteolin-di-*O*-glucuronide (**17**); and *C*-glycosides—quercetin- and luteolin-*C*-deoxyhexoside-*C*-hexosides (**12** and **14**, respectively). Quercetin, luteolin, apigenin, and kaempferol derivatives (free aglycones and their glycosides) were previously reported as abundant constituents in the *Origanum* genus [11,15,18,34]. Several terpenes, such as the three monoterpenes thymoquinol-*O*-hexoside (**10**), *p*-menth-1-ene-3,4-diol-*O*-hexoside (**16**), and carvone (**42**), as well as the two diterpenes carnosol (**47**) and dehydrocarnosol (**48**), were also tentatively identified in the oregano extracts. These compounds were previously reported in various *Origanum* species [14]. Tuberonic acid (**11**) and its glycoside (**7**) and trihydroxyoctadecadienoic acid I (**36**), hydroxyoctadecatrienoic acid (**49**), and hydroxyoctadecadienoic acid (**51**) were the characteristic fatty acids in the WE, SE, and TE of oregano. From a qualitative point of view, there were no significant differences between the three oregano extracts. Some polar compounds, glycosides of phenolic acids or monoterpenes (**3**, **4**, **6,** and **10**), were not detected in TE. In contrast, some hydrophobic metabolites, such as diterpenes and fatty acids (**47**, **49**, **50**, and **51**), were absent in the WE.

A total of 25 specialized metabolites were labeled in the basil extracts (Table 3). As mentioned above for the thyme and oregano extracts, danshensu (**2**), hydroxybenzoic acid (**4**), caffeic acid (**9**), salvianolic acid H (**21**), salvianolic acid D (**25**), rosmarinic acid (**26**), and 3,4,4′-trihydroxy-5,5′-diisopropyl-2,2′-dimethylbiphenyl (**52**) were also annotated as phenolic compounds in WE, SE, and TE of oregano. In addition, caftaric acid (**5**), fertaric acid (**13**), cichoric acid (**29**), and salvianolic acid isomer (**34**) were also evidenced. Most of these phenolic metabolites were previously reported in *Ocimum* species by LC-MS analyses [28,40]. The group of flavonoids was represented by eight derivatives, such as free aglycones: luteolin (**35**), cirsimaritin (**38**), ladanein (**40**), and cirsilineol (**45**); *O*-glycosides—quercetin-*O*-pentoside-*O*-hexoside (**19**), quercetin-*O*-hexoside II (**22**), and luteolin-*O*-deoxyhexoside-*O*-hexoside (**24**); and *C*-glycosides: luteolin-*C*-deoxyhexoside-*C*-hexoside (**14**). Flavonoids with similar structures were previously documented in the *Ocimum* genus [22,28,40]. Lastly, four fatty acids (trihydroxyoctadecadienoic acid I, **36**, hydroperoxyoctadecadienoic acid, **46**, hydroxyoctadecatrienoic acid, **49**, and hydroxyoctadecadienoic acid **51**), quinic acid (**1**), and roseoside (**8**) were also spotted in the obtained basil extracts. The differences observed between WE, SE, and TE were not significant. For instance, caffeic acid (**9**), a few flavonoids (**19**, **22**, **24**, **35**, and **40**), and salvianolic acid D (**25**) were absent from the TE of oregano. In contrast, some lipophilic fatty acids (**46** and **51**) were not retrieved in the WE.

**Table 3:** LC-HRMS/MS profile of extracts obtained from thyme, oregano, and basil.

No	Compound	Class	T_R_ (min)	[M–H]^−^ (*m*/*z*)	MF	HRMS/MS (*m*/*z*)	Ref.	Thyme	Oregano	Basil
1	Quinic acid *	Organic acid	1.8	191.0575	C_7_H_12_O_6_	177.0423, 159.0319, 129.0207	[38]	W,S,T	W,S,T	W,S,T
2	Danshensu	Phenolic	5.4	197.0445	C_9_H_10_O_5_	179.0340, 135.0439, 123.0443	[34]	W,S,T	W,S,T	W,S,T
3	Hydroxybenzoic acid-*O*-hexoside	Phenolic	7.8	299.0764	C_13_H_16_O_8_	137.0239	[41]	W,S	W,S	–
4	Hydroxybenzoic acid	Phenolic	9.4	137.0244	C_7_H_6_O_3_	108.0185	[1]	W,S,T	W,S	W,S,T
5	Caftaric acid	Phenolic	11.8	311.0374	C_13_H_12_O_9_	179.0309, 149.0059, 135.0423	[22,40]	–	–	W,S,T
6	Caffeic acid-*O*-hexoside	Phenolic	12.3	341.0351	C_15_H_18_O_9_	179.0351, 135.0447	[15]	W,S	W,S	–
7	Tuberonic acid-*O*-hexoside	Fatty acid	13.7	387.1709	C_18_H_8_O_9_	207.0956; 101.0232	[14]	–	W,S,T	–
8	Roseoside	Phenolic	13.4	385.1883	C_19_H_30_O_8_	223.1321, 205.1197, 179.0539, 153.0914	[14]	–	–	W,S,T
9	Caffeic acid *	Phenolic	13.9	179.0357	C_9_H_8_O_4_	135.0454, 107.0505	[22,34,38]	W,S,T	–	W,S
10	Thymoquinol-*O*-hexoside	Monoterpene	14.3	327.1457	C_16_H_24_O_7_	165.0842, 149.0610, 101.0249	[14,42]	–	W,S	–
11	Tuberonic acid	Fatty acid	16.1	225.1150	C_12_H_18_O_4_	207.0994, 165.0926, 147.0799, 135.0799	[14]	W,S,T	W,S,T	–
12	Quercetin-*C*-deoxyhesoide-*C*-hexoside	Flavonoid	16.7	609.1468	C_27_H_30_O_16_	519.1140, 489.1047, 429.0832, 399.0724, 369.0621	[5,34]	–	W,S,T	–
13	Fertaric acid	Phenolic	17.3	325.0566	C_14_H_14_O_9_	193.0506, 135.0371	[22]	–	–	W,S,T
14	Luteolin-*C*-deoxyhexoside-*C*-hexoside	Flavonoid	18.7	593.1521	C_27_H_30_O_15_	503.1198, 473.1088, 383.0771, 353.0673	[34]	W,S,T	W,S,T	W,S,T
15	Aromadendrin-*O*-hexoside	Flavonoid	19.5	449.1102	C_21_H_22_O_11_	287.0555, 151.0040, 135.0242	[38]	T	–	–
16	*p*-Menth-1-ene-3,4-diol-*O*-hexoside	Monoterpene	20.8	331.1761	C_16_H_28_O_7_	179.0563, 161.0455, 143.0342, 119.0350	[14,42]	W,S,T	W,S,T	–
17	Luteolin-di-*O*-glucuronide	Flavonoid	21.8	637.1207	C_31_H_26_O_15_	351.0663, 285.0488, 193.0405, 175.0296	[14]	–	W,S,T	–
18	Quercetin-*O*-hexoside I	Flavonoid	21.9	463.0938	C_21_H_20_O_12_	301.0356, 300.0286	[14,15]	W,S,T	–	–
19	Quercetin-*O*-pentoside-*O*-hexoside	Flavonoid	22.3	595.1301	C_26_H_28_O_16_	300.0265, 271.0251, 255.0289, 197.0452, 151.0032, 135.0438	[14]	–	–	W,S
20	Taxifolin	Flavonoid	22.7	303.0511	C_15_H_12_O_7_	285.0338, 275.0543, 259.0598, 177.0181, 125.0235	[14]	T	W,S,T	–
21	Salvianolic acid H	Phenolic	23.2	537.0992	C_27_H_22_O_12_	493.1259, 339.0598, 313.0792, 295.0686, 269.0892, 197.0506, 179.0392	[22]	–	W,S,T	W,S,T
22	Quercetin-*O*-hexoside II	Flavonoid	23.9	463.0988	C_21_H_20_O_12_	301.0406, 271.0284, 179.0017, 151.0062	[14,15]	W,S,T	–	W,S
23	Gallocatechin *	Flavonoid	24.5	305.0745	C_15_H_14_O_7_	225.1161	[14]	W,S,T	W,S,T	–
24	Luteolin-*O*-deoxyhexoside-*O*-hexoside	Flavonoid	24.7	593.1551	C_27_H_30_O_15_	285.0406, 255.0282, 227.0314, 151.0032	[14,15]	–	–	W,S
25	Salvianolic acid D	Phenolic	25.4	417.0936	C_20_H_18_O_10_	399.0816, 373.1028, 237.0463, 197.0507, 175.0448	[43]	–	W,S,T	W,S
26	Rosmarinic acid *	Phenolic	26.7	359.0829	C_18_H_16_O_8_	197.0483, 179.0369, 135.0461, 123.0465	[14,15,22,38]	W,S,T	W,S,T	W,S,T
27	Salvianolic acid B	Phenolic	27.5	717.1659	C_36_H_30_O_16_	537.1194, 519.1105, 493.1329, 475.1197, 359.0867, 339.0591, 321.0500, 197.0504, 179.0398	[22]	–	W,S,T	–
28	Salvianolic acid A	Phenolic	27.8	493.1286	C_26_H_22_O_10_	313.0806, 295.0693, 185.0293	[38]	–	W,S,T	–
29	Cichoric acid	Phenolic	28.3	473.0701	C_22_H_18_O_12_	311.0341, 293.0243, 179.0311, 149.0056	[44]	–	–	W,S,T
30	Thymol-*O*-hexoside	Monoterpene	28.5	311.1476	C_16_H_24_O_6_	197.0453, 161.0256, 149.0970	[14,42]	W,S,T	–	–
31	Salvianolic acid I	Phenolic	28.8	537.1165	C_27_H_22_O_12_	493.1131, 359.0766, 313.0702, 295.0606, 197.0443, 179.0342	[22]	–	W,S,T	–
32	Eriodictyol	Flavonoid	29.4	287.0573	C_15_H_12_O_6_	151.0033, 127.0332	[14]	W,S,T	–	–
33	Salvianolic acid E	Phenolic	29.7	717.1654	C_36_H_30_O_16_	537.1166, 519.1077, 339.0607, 321.0498, 295.0698, 197.0505	[22]	–	W,S,T	–
34	Salvianolic acid A isomer	Phenolic	30.2	493.1103	C_26_H_22_O_10_	313.0654, 295.0554, 185.0202,	[34]	–	–	W,S,T
35	Luteolin *	Flavonoid	31.1	285.0439	C_15_H_10_O_6_	267.0320, 199.0413, 175.0413, 151.0046, 133.0303	[3,14,18]	W,S,T	–	W,S
36	Trihydroxyoctadecadienoic acid I	Fatty acid	32.2	327.2204	C_18_H_32_O_5_	229.1451, 171.1020	[14]	W,S,T	W,S,T	W,S,T
37	Apigenin *	Flavonoid	32.8	271.0619	C_15_H_10_O_5_	177.0183, 151.0030, 119.0496	[3]	–	W,S,T	–
38	Cirsimaritin	Flavonoid	33.0	313.0740	C_17_H_14_O_6_	161.0246, 151.0399, 133.0297	[14]	W,S,T	–	W,S,T
39	Trihydroxyoctadecenoic acid I	Fatty acid	33.9	329.2336	C_18_H_34_O_5_	229.1448, 211.1337, 171.1026	[14,40]	W,S,T	–	–
40	Ladanein	Flavonoid	34.2	313.0718	C_17_H_14_O_6_	269.0828, 161.0246, 151.0396, 133.0290	[45]	–	–	W,S
41	Kaempferol *	Flavonoid	34.4	285.0387	C_15_H_10_O_6_	255.0289, 239.0330, 185.0580, 151.0023 117.0332	[3,34]	–	W,S,T	–
42	Carvone	Monoterpene	35.2	165.0910	C_10_H_14_O_2_	149.0608, 135.0441, 107.0486	[40]	–	W,S,T	–
43	Trihydroxyoctadecadienoic acid II	Fatty acid	35.5	327.2204	C_18_H_32_O_5_	229.1443, 201.1133, 171.1007	[14]	W,S,T	–	–
44	Pebrellin	Flavonoid	39.1	373.0990	C_19_H_18_O_8_	358.0702, 343.0469, 328.0224, 300.0285, 285.0056	[14]	W,S,T	–	–
45	Cirsilineol	Flavonoid	37.4	343.0847	C_18_H_16_O_7_	328.0601, 313.0367, 298.0134, 285.0417, 270.0183,	[14]	W,S,T	–	W,S,T
46	Hydroperoxyoctadecadienoic acid	Fatty acid	40.6	311.2230	C_18_H_32_O_4_	293.2130, 275.2039, 223.1704	[14]	W,S,T	–	S
47	Carnosol	Diterpene	42.2	329.1764	C_22_H_26_O_4_	314.1506, 299.1286, 271.0977	[14,34]	W,S,T	S,T	–
48	Dehydrocarnosol	Diterpene	45.5	327.1615	C_22_H_24_O_4_	299.1680, 284.1334, 269.1113	[14]	W,S,T	W,S,T	–
49	Hydroxyoctadecatrienoic acid	Fatty acid	46.7	293.2125	C_18_H_30_O_3_	275.2021, 183.1392	[14]	W,S,T	S,T	W,S,T
50	4′-Hydroxy-5,5′-diisopropyl-2,2′-dimethyl-3,4-biphenylquinone	Phenolic	48.6	311.1663	C_20_H_24_O_3_	283.1711, 268.1130, 253.1164, 240.1167, 225.0946, 187.1115	[39]	S,T	S,T	–
51	Hydroxyoctadecadienoic acid	Fatty acid	49.4	295.2296	C_18_H_32_O_3_	277.2133, 195.1391, 171.1030	[14]	S,T	S,T	S,T
52	3,4,4′-Trihydroxy-5,5′-diisopropyl-2,2′-dimethylbiphenyl	Phenolic	51.5	313.1810	C_20_H_26_O_3_	297.1513, 283.1357, 270.1264, 255.1032	[39]	W,S,T	W,S,T	W,S,T

MF, molecular formula; S, spent extract; T, total extract; T_R_, retention time; W, residual water extract; * Confirmed by standard.

**Figure 1:**
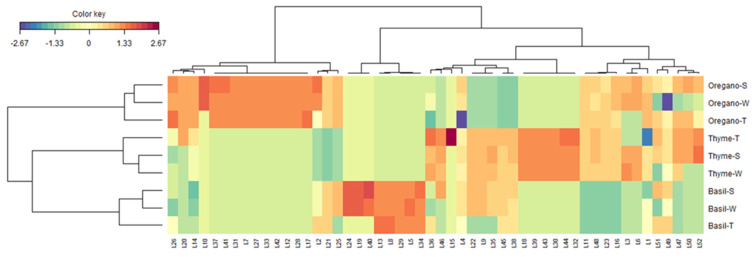
Clustered image map (Red color: high bioactivity. Blue color: low bioactivity) on LC-HRMS/MS derived dataset. For compound numbers, refer to Table 3.

As can be seen from the above discussion, numerous specialized metabolites were simultaneously found in all three Lamiaceae species. The heatmap approach was used to identify the key molecules unique to thyme, oregano, and basil. Firstly, as shown in Figure 1, the extracts were separated according to the species. Generally, oregano samples were abundant in rosmarinic acid (L26), taxifolin (L20), luteolin-*C*-deoxyhexoside-*C*-hexoside (L14), apigenin (L37), kaempferol (L41), salvianolic acid I (L31), tuberonic acid-*O*-hexoside (L7), salvianolic acid B (L27), salvianolic acid E (L33), carvone (L42), quercetin-*C*-deoxyhesoide-*C*-hexoside (L12), salvianolic acid A (L28), luteolin-di-*O*-glucuronide (L17). In addition, a high concentration of thymoquinol-*O*-hexoside (L10) and danshensu (L2) were observed in the spent material and residual extracts of oregano. Thyme samples were also characterized by various constituents, among which quercetin-*O*-hexoside I (L18), trihydroxyoctadecenoic acid I (L39), trihydroxyoctadecadienoic acid II (L43), cymenol-*O*-hexoside (L30), pebrellin (L44) and eriodictyol (L32) predominated. Similarly, a high amount of trihydroxyoctadecadienoic acid I (L36) and aromadendrin-O-hexoside (L15) was detected in the total extract, while a significant level of 3,4,4′-trihydroxy-5,5′-diisopropyl-2,2′-dimethylbiphenyl (L52) was found in the spent extract. In the case of basil, fertaric acid (L13), roseoside (L8), cichoric acid (L29), caftaric acid (L5), and salvianolic acid A isomer (L34) were found to be the main distinctive constituents. In addition, the spent and residual water extracts were richer in luteolin-*O*-deoxyhexoside-*O*-hexoside (L24), quercetin-*O*-pentoside-*O*-hexoside (L19), and ladanein (L40).

In conclusion, the by-products of the hydrodistillation of the three Lamiaceae herbs, residual water and spent material extracts, can be regarded as rich sources of bioactive metabolite, mainly phenolic compounds. Compared with the total (unspent material) extracts, no groundbreaking qualitative differences in the phytochemical profiles were observed for the by-product extracts. Generally, the spent extracts showed the highest abundance of phytochemicals; several possible theories can be formulated. On the one hand, compounds that might be found in small amounts in the original (unspent) plant materials and are transferred neither into the hydrodistillate nor into the water used for hydrodistillation may become more accessible to the subsequent solvent extraction of the spent plant materials. On the other hand, following the long exposure of the plant material to boiling water, a cell permeation effect can be assumed, favoring the subsequent extraction of the metabolites or even the transfer of medium or low polarity molecules into the hydrodistillation water. In addition, due to the harsh hydrodistillation conditions (high temperatures and long exposure times), the formation of phenolic artifacts in the residual water and spent extracts cannot be excluded.

### 3.3. Total Phenolic and Flavonoid Content of Total, Spent, and Residual Water Extracts

Phenolic compounds are one of the most popular class of phytochemicals that are gaining increasing interest due to their wide range of biological activities [46]. In this sense, estimating the phenolic content could provide the first clues about their biological potential. In this section, the total phenolic and flavonoid content of the total, spent, and residual water extracts of thyme, oregano, and basil were determined, with the results summarized in Table 4. In basil, the highest total phenolic content was found in the spent extract with 93.66 mg GAE/g, followed by the residual water (63.57 mg GAE/g) and total (58.85 mg GAE/g) extracts. Regarding thyme, the residual water extract had the highest concentration of total phenolics (88.69 mg GAE/g). However, the spent and total extracts of thyme had lower values. In oregano, the phenolic levels were reported as 115.71, 113.34, and 110.35 mg GAE/g for total, spent, and residual water extract, respectively. Concerning the total flavonoid content, the spent extracts of all three species had higher concentrations than the total and residual water extracts. The highest level of total flavonoid was determined in the spent extracts of oregano (34.88 mg RE/g), followed by thyme (28.16 mg RE/g) and basil (27.59 mg RE/g). The extraction process subsequent to hydrodistillation significantly increased the total flavonoid concentration. Based on these findings, the hydrodistillation process may destroy the cell wall, allowing the methanol to enter the cell faster and more efficiently during the extraction process. Some antioxidant compounds were not dissolved in boiling water in a previous study; thus, extraction with polar solvents such as methanol or ethanol could help extract more compounds from materials after hydrodistillation [47]. In line with our findings, several researchers have reported that post-hydrodistillation extraction increased flavonoid levels [48,49].

### 3.4. Antioxidant Activity of Essential Oils, Total, Spent, and Residual Water Extracts

Antioxidants are thought to protect against the onslaught of free radicals, which are the primary cause of the progression of severe health conditions, such as Alzheimer’s, diabetes, and cancer. In this regard, identifying new raw materials as sources of antioxidants has become one of the most popular topics within the scientific community [50]. The EOs and extracts of thyme, oregano, and basil were tested in various antioxidant assays (Table 5). In the DPPH assay, the extracts showed a more significant activity than EOs. Concerning the thyme extracts, the highest activity was noticed in the residual water extract (DPPH: 121.00 mg TE/g; ABTS: 150.09 mg TE/g). The most active oregano samples were the spent (266.59 mg TE/g) and total extracts (347.67 mg TE/g) for DPPH and ABTS, respectively. In basil, the highest free radical scavenging ability was determined in spent extract (DPPH: 135.88 mg TE/g; ABTS: 144.57 mg TE/g). When all extracts were evaluated together, oregano extracts were more active than the corresponding basil and thyme extracts. As can be seen in Table 5, the EOs showed a more substantial CUPRAC power than the extracts; the EO of oregano was the most active sample at 1792.32 mg TE/g. In the CUPRAC assay, the spent extracts of basil and oregano displayed the highest capacity, while the residual water of thyme was the most active. Basil and thyme EOs were the most potent in the FRAP assay, whereas oregano’s spent extract was the most effective. The EO of basil (31.24 mmol TE/g) was the most active in the PBD assay, followed by the EOs of thyme (10.14 mmol TE/g) and oregano (5.74 mmol TE/g). These findings are consistent with the CUPRAC and FRAP results. Overall, the results of the extracts from all samples were almost the same in the free radical scavenger and reducing power tests. Some compounds in the extracts, including caftaric acid, roseoside, fertaric acid, and cichoric acid, were strongly correlated with antioxidant activity (Table S1). Consistent with our findings, several researchers have described these compounds as powerful antioxidants [51,52,53,54]. In addition, Table S2 shows the correlation between EO compounds and antioxidant properties. For example, *cis*-terpineol and thymol methyl ester strongly correlated with DPPH scavenging ability, while limonene and camphene mainly contributed to the FRAP power.

In contrast to other antioxidant assays, the highest metal chelating propensities were found in the residual water extracts of the three species (10.74–17.50 mg EDTAE/g). As can be seen in Table S1, no compounds correlated with the observed metal chelating ability. In this sense, the activity can be explained by the presence of non-phenolic chelating agents, including polysaccharides or peptides. Regarding the metal chelating potential of EOs, only the EO from basil was active with 1.34 mg EDTAE/g, but others showed no chelating ability. The spent extracts from the tested Lamiaceae species (especially basil and oregano) contained significant antioxidant properties [55,56,57]. Consequently, the byproducts of post-distillation can be considered rich sources of natural antioxidants with potential pharmaceutical and nutraceutical applications.

### 3.5. Enzyme Inhibitory Activity of Essential Oils, Total, Spent, and Residual Water Extracts

Inhibiting key enzymes associated with the pathologies of global health issues may benefit disease treatment. For example, amylase and glucosidase are essential enzymes for hydrolyzing carbohydrates, and when inhibited, diabetic patients’ blood glucose levels can be regulated [58]. Similarly, AChE breaks down acetylcholine in the synaptic cleft under normal conditions. If AChE is inhibited in Alzheimer’s patients, the level of acetylcholine in the synaptic cleft can rise, thereby enhancing the cognitive functions in the patients [59]. In this sense, several compounds have been chemically designed as inhibitor drugs used in clinical practice. Their long-term use, however, resulted in adverse health effects such as gastrointestinal disturbances and hepatotoxicity [60]. This study evaluated the enzyme inhibitory properties of thyme, oregano, and basil against cholinesterases, tyrosinase, amylase, and glucosidase (Table 6). Basil and thyme EOs had the highest AChE inhibitory activity (basil: 3.29 mg GALAE/g; thyme: 1.80 mg GALAE/g), followed by spent, total, and residual water extracts.

Regarding the AChE inhibitory effects of oregano samples, the order of potency was total extract > EO > spent extract > residual water. Surprisingly, only the EOs showed inhibitory effects on BChE. The best BChE inhibitory effect was observed with EOs of basil, with 1.28 mg GALAE/g, followed by oregano and thyme. In summary, the tested EOs had good potential as cholinesterase inhibitors. A strong correlation between several EOs constituents (e.g., α-pinene, cis-α-terpineol, and *p*-cymene) and their cholinesterase inhibitory properties was noticed (Table S4). In the literature, several authors pointed out that the EOs have significant cholinesterase inhibiting effects [61,62,63].

In terms of the tyrosinase inhibitory activity, the best capacity was found among the EOs; for the extracts, the following decreasing order of activity was noticed: spent > total > residual water. In the correlation analysis, some compounds in the extracts and EOs might contribute to the observed tyrosinase inhibitory properties (Tables S3 and S4). Concerning the anti-diabetic-related properties, the anti-amylase activity of the EOs was succeeded by that of the spent, total, and residual water extracts. Interestingly, no EO showed any inhibitory effects on glucosidase. The highest glucosidase inhibitory values were recorded in the spent extracts, followed by the total and residual water extracts. Several previous studies were conducted on the enzyme inhibitory properties of basil, thyme, and oregano EOs or extracts. The literature indicates that these species had significant potential as natural enzyme inhibitors as well as antioxidants [64,65,66,67,68,69,70,71]. Furthermore, our findings shed light on the valuable activity of post-distillation by-products, which can be used to develop novel enzyme inhibition drugs. These findings could also aid in the recycling of plant materials following EO extraction.

### 3.6. Multivariate Analysis

Principal component analysis (PCA) and heatmapping are widely used to analyze complex chemical and biological data. While PCA is used to reduce the dimensionality of high-dimensional data, heatmaps represent a data matrix in which rows and/or columns of the matrix are clustered to allow the visualization of the values in the cells by using a color gradient [72].

**Figure 2:**
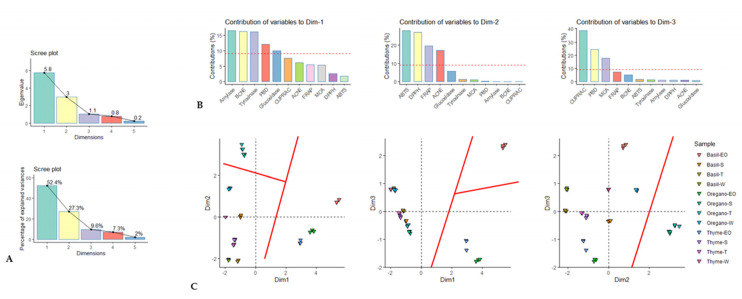
Principal component analysis. (**A**) Eigenvalue and percentage of explained variance of each dimension. (**B**) Contribution of biological activities on the principal components of PCA. (**C**) Scatter plot showing the distribution of the samples in the factorial plan derived from the three retained principal components.

In this study, PCA was initially used to explain the differences between the samples and obtain information on bioactivity. The results of the PCA are given in Figure 2A–C. Dimension 1 accounted for 52.4% of the total variance and was primarily associated with amylase, BChE, tyrosinase, and, to a lesser extent, PBD and glucosidase. Dimension 2 accounted for 27.3% of the total variance and was determined primarily by ABTS, DPPH, FRAP, and AChE, whereas dimension 3 accounted for 9.6% of the total variance and was defined by CUPRAC, PBD, and MCA. These results suggest that the extracts are separated along each dimension according to the bioactivities that characterize them. These three dimensions were retained because they had eigenvalues greater than 1. The next step was to explore the different scatter plots formed by the three retained dimensions. In the scatter plot Dim-1 vs. Dim-2, the EOs of the three species were separated from the other extracts. In addition, the spent and total extracts of oregano were more distant from the other extracts. In the scatter plot Dim-1 vs. Dim-3, the EO of basil stood out from the EOs of thyme and oregano. This trend was confirmed in the scatter plot Dim-2 vs. Dim-3.

**Figure 3:**
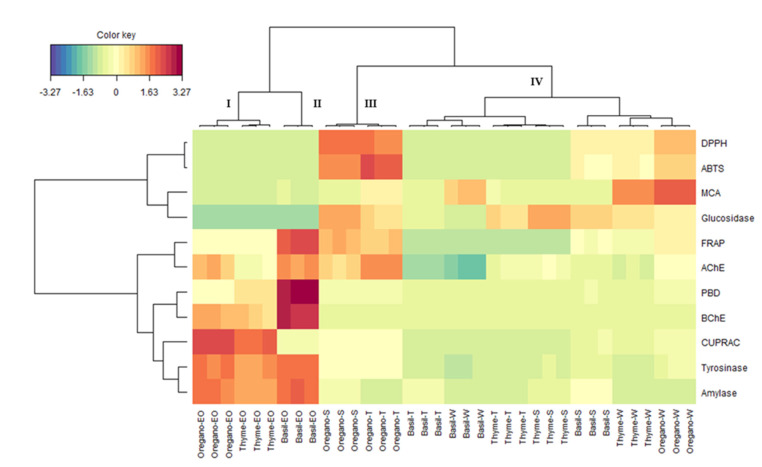
Clustered image map (Red color: high bioactivity. Blue color: low bioactivity) on biological activity dataset.

Next, a Heatmap analysis was performed to better represent the various clusters and visualize the differences in bioactivities between each cluster. As shown in Figure 3, the extracts were separated into four distinct groups. Oregano EO and thyme EO were in cluster I, while basil EO represented cluster II. Cluster I showed stronger anti-tyrosinase and anti-amylase activities, as also demonstrated by cluster II. In addition, cluster I exhibited remarkable CUPRAC activity, while cluster II showed the highest anti-BChE, PBD, and FRAP activities.

Furthermore, it was noticed that the EOs of clusters I and II were segregated from the other extracts, which represented clusters III and IV. Hence, spent and total extracts of oregano showed a similar bioactivity profile and were placed in cluster III. This cluster exhibited higher ABTS and DPPH activities. Lastly, the remaining extracts were grouped in cluster IV. Overall, the extracts of this cluster were characterized by low bioactivity, except for the residual water extracts of thyme and oregano, which showed significant chelating activity. The merged result showed that the heatmap conclusion agreed with the PCA observation. Accordingly, we can suggest that PCA coupled with the heatmap was suitable for discriminating the study samples.

## 4. Conclusions

In this work, two raw extracts (EOs obtained by hydrodistillation and total extracts) and two post-distillation by-products (spent material and residual water extracts) of thyme, oregano, and basil were phytochemically and biologically evaluated. More than 90 volatile compounds (hydrocarbon monoterpenes, oxygenated monoterpenes, hydrocarbon sesquiterpenes, oxygenated sesquiterpenes, etc.) were identified by GC-MS in the essential oils of the three Lamiaceae species. Around 50 specialized metabolites belonging to various phytochemical classes, such as phenolic and organic acids (e.g., quinic acid, hydroxybenzoic, caffeic acid, salvianolic acids, etc.), flavonoids (luteolin, kaempferol, quercetin and their *O*- and *C*-glycosides), diterpenes (e.g., carnosol), and fatty acids, were assigned with the use of LC-HRMS/MS in the solvent-based extracts. The Heatmap analysis revealed that the spent extracts showed a higher abundance of phenolic phytochemicals than the total or residual water extracts. Concerning the biological evaluations, all tested samples displayed strong antioxidant potential; the EOs were the most active metal reducing agents, whereas the spent and total extracts were the best scavengers of DPPH and ABTS. In addition, the EOs also displayed the highest cholinesterase and tyrosinase inhibitory properties, while the highest anti-glucosidase effects were recorded for the spent extracts.

Overall, the results of the current study provide new and insightful contributions to the continuously expanding body of knowledge regarding the potential valorization of post-distillation by-products as a low-cost source of high-value constituents for the food, pharmaceutical, and cosmetics industries. Furthermore, this can constitute a starting point for finding new ways of exploiting the large amounts of waste produced worldwide by the EO industry, with beneficial environmental, technological, and economic advantages.

## Figures and Tables

**Table 1:** Extraction yields of thyme, oregano, and basil.

Material	Yield EO [mL/%wt]	Yield WE [%wt]	Yield SE [%wt]	Yield TE [%wt]
Thyme	2.3 ± 0.1	29.2 ± 1.1	9.3 ± 0.8	10.5 ± 1.0
Oregano	4.3 ± 0.1	42.2 ± 1.4	9.3 ± 0.5	16.0 ± 2.2
Basil	1.4 ± 0.2	32.8 ± 1.4	11.0 ± 1.4	18.4 ± 2.0

EO, essential oil; SE, spent extract; TE, total extract; WE, residual water extract; data are presented as average ± standard deviation of two repeated experiments.

**Table 2:** GC-MS profile of the essential oils obtained from thyme, oregano, and basil.

No.	Compound	LRI ^a^	Thyme (%) ^b^	Oregano (%) ^b^	Basil (%) ^b^
1	Methyl 2-methylbutanoate	785	0.18 ± 0.01	Nd	Nd
2	α-Phellandrene	927	0.62 ± 0.03	0.22 ± 0.01	Nd
3	α-Pinene	935	1.08 ± 0.03	0.34 ± 0.02	0.29 ± 0.03
4	Camphene	952	0.59 ± 0.02	0.10 ± 0.01	0.05 ± 0.01
5	Sabinene	974	Nd	Nd	0.16 ± 0.01
6	β-Pinene	980	1.33 ± 0.04	0.42 ± 0.02	0.53 ± 0.05
7	3-Octanone	985	0.06 ± 0.00	0.08 ± 0.00	Nd
8	β-Myrcene *	989	0.83 ± 0.02	0.42 ± 0.02	0.19 ± 0.01
9	3-Octanol	996	0.14 ± 0.01	0.04 ± 0.00	Nd
10	3-Thujene	1007	0.10 ± 0.01	0.07 ± 0.00	Nd
11	3-Carene	1009	0.08 ± 0.00	Nd	Nd
12	α-Terpinene	1018	1.16 ± 0.03	0.71 ± 0.02	0.09 ± 0.00
13	*p*-Cymene	1027	**25.70 ± 0.39**	**6.71 ± 0.11**	0.06 ± 0.00
14	Limonene *	1031	0.48 ± 0.01	0.27 ± 0.01	0.23 ± 0.02
15	Eucalyptol	1034	1.01 ± 0.02	0.03 ± 0.0	**5.46 ± 0.48**
16	*trans*-β-Ocimene	1045	Nd	Nd	0.05 ± 0.01
17	*cis*-α-Ocimene	1047	Nd	Nd	0.08 ± 0.08
18	γ-Terpinene	1060	**4.89 ± 0.10**	2.87 ± 0.03	0.16 ± 0.01
19	4-Pentenyl butyrate	1065	0.05 ± 0.00	Nd	Nd
20	*cis*-α-Terpineol	1072	0.28 ± 0.01	0.09 ± 0.00	0.18 ± 0.01
21	α-Terpinolene	1087	0.14 ± 0.01	0.09 ± 0.01	0.18 ± 0.03
22	*p*-Cymenene	1091	0.09 ± 0.00	Nd	Nd
23	Linalool *	1099	2.96 ± 0.05	0.15 ± 0.01	**17.70 ± 1.00**
24	*trans*-5-Caranol	1101	Nd	0.06 ± 0.00	Nd
25	Fenchyl alcohol	1121	Nd	Nd	0.04 ± 0.00
26	*cis*-*p*-Menth-2-en-1-ol	1127	0.10 ± 0.00	0.04 ± 0.00	Nd
27	*trans-p*-Menth-2-en-1-ol	1145	0.10 ± 0.00	0.04 ± 0.00	Nd
28	Camphor	1150	0.40 ± 0.01	Nd	0.52 ± 0.03
29	*cis*-Terpin hydrate	1173	Nd	Nd	0.19 ± 0.01
30	Borneol	1176	1.61 ± 0.02	0.83 ± 0.01	0.31 ± 0.01
31	Terpinen-4-ol	1183	1.25 ± 0.02	0.98 ± 0.02	0.68 ± 0.03
32	*p*-Cymen-8-ol	1188	0.12 ± 0.01	0.02 ± 0.00	Nd
33	Estragole	1191	Nd	Nd	**19.62 ± 0.80**
34	*trans*-α-Terpineol	1197	0.43 ± 0.02	0.23 ± 0.00	Nd
35	Dihydrocarvone	1199	Nd	0.27 ± 0.01	Nd
36	Octyl acetate	1201	Nd	Nd	0.06 ± 0.01
37	*cis*-Geraniol	1219	Nd	Nd	0.06 ± 0.02
38	Thymol methyl ether	1224	1.44 ± 0.02	Nd	Nd
39	Isothymol methyl ether	1235	1.06 ± 0.01	0.60 ± 0.01	Nd
40	*d*-Darvone	1245	Nd	0.03 ± 0.00	0.05 ± 0.00
41	*trans*-Geraniol	1247	0.16 ± 0.01	Nd	0.29 ± 0.01
42	*m*-Cymene	1273	0.08 ± 0.01	Nd	Nd
43	Thymol isomer	1281	0.59 ± 0.01	0.07 ± 0.01	Nd
44	Bornyl acetate	1286	Nd	Nd	0.68 ± 0.01
45	Thymol	1293	**40.26 ± 0.36**	**9.40 ± 0.30**	0.04 ± 0.00
46	Carvacrol	1292	**4.48 ± 0.06**	**69.93 ± 0.75**	Nd
47	(Z)-Methyl cinnamate	1297	Nd	Nd	2.02 ± 0.04
48	Isoeugenol	1322	Nd	0.04 ± 0.01	Nd
49	2-Hydroxycineole acetate	1336	Nd	Nd	0.09 ± 0.00
50	Eugenol	1350	0.10 ± 0.01	Nd	3.55 ± 0.99
51	Isobornyl propionate	1378	0.10 ± 0.01	Nd	Nd
52	Copaene	1380	0.10 ± 0.0	Nd	0.20 ± 0.01
53	α-Farnesene	1390	0.07 ± 0.01	Nd	Nd
54	(*E*)-Methyl cinnamate	1387	Nd	Nd	**12.06 ± 0.27**
55	β-Elemene	1394	Nd	Nd	0.99 ± 0.06
56	Methyl eugenol	1398	Nd	Nd	2.78 ± 0.07
57	(*E*)-α-Bergamotene	1417	Nd	Nd	0.10 ± 0.01
58	Caryophyllene *	1428	2.26 ± 0.08	1.75 ± 0.07	1.16 ± 0.05
59	(*Z*)-α-Bergamotene	1437	0.03 ± 0.00	Nd	**7.11 ± 0.13**
60	4-*t*-Butyl-pyrocatechol	1442	0.17 ± 0.03	0.04 ± 0.00	Nd
61	γ-Elemene	1447	Nd	0.04 ± 0.01	0.04 ± 0.00
62	β-Farnesene	1453	Nd	Nd	0.39 ± 0.02
63	Cedrene	1457	Nd	Nd	0.02 ± 0.00
64	Humulene *	1464	0.08 ± 0.01	0.23 ± 0.02	0.92 ± 0.04
65	Nerol acetate	1467	0.13 ± 0.01	Nd	Nd
66	β-Cubenene	1470	Nd	Nd	0.49 ± 0.03
67	α-Himalachene	1474	Nd	Nd	0.05 ± 0.00
68	α-Huaiene	1481	0.25 ± 0.01	0.05 ± 0.01	0.27 ± 0.02
69	Germacrene D	1489	Nd	Nd	2.18 ± 0.10
70	β-Selinene	1498	0.08 ± 0.01	0.02 ± 0.00	0.29 ± 0.02
71	α-Selinene	1504	0.12 ± 0.01	Nd	0.59 ± 0.03
72	β-Bisabolene	1508	0.12 ± 0.01	1.32 ± 0.09	0.95 ± 0.05
73	γ-Cadinene	1520	0.30 ± 0.02	0.05 ± 0.01	3.46 ± 0.19
74	β-Cadinene	1524	0.29 ± 0.02	0.11 ± 0.01	Nd
75	δ-Cadinene	1528	0.15 ± 0.02	0.05 ± 0.01	0.68 ± 0.04
76	α-Bisabolene	1543	Nd	Nd	0.84 ± 0.05
77	(*E*)-Farnesene epoxide	1548	Nd	Nd	0.09 ± 0.01
78	Nerolidol	1562	Nd	Nd	0.26 ± 0.02
79	Globulol	1580	Nd	Nd	0.17 ± 0.01
80	Spathulenol	1586	0.04 ± 0.01	0.07 ± 0.01	0.72 ± 0.07
81	β-Caryophyllene oxide *	1593	1.01 ± 0.08	0.94 ± 0.08	0.41 ± 0.05
82	Aromadendrene oxide	1603	Nd	Nd	0.10 ± 0.01
83	*cis*-(*Z*)-α-Bisabolene epoxide	1611	Nd	Nd	0.13 ± 0.01
84	*trans*-(*E*)-α-Bisabolene epoxide	1620	Nd	0.07 ± 0.01	Nd
85	Cubenol	1623	0.09 ± 0.01	Nd	0.74 ± 0.06
86	γ-Eudesmol	1633	0.11 ± 0.01	Nd	Nd
87	*trans*-(*Z*)-α-Bisabolene epoxide	1644	Nd	Nd	0.53 ± 0.07
88	α-Cadinol	1650	0.29 ± 0.02	Nd	**5.64 ± 0.76**
89	β-Eudesmol	1663	0.07 ± 0.01	Nd	0.64 ± 0.01
90	*allo*-Aromadendrene epoxide	1678	0.11 ± 0.01	0.04 ± 0.01	0.19 ± 0.03
91	α-Bisabolol *	1692	Nd	Nd	0.28 ± 0.05
92	Ledene alcohol	1700	Nd	Nd	0.11 ± 0.03
93	Ledene oxide	1717	Nd	Nd	0.12 ± 0.03
94	Isoaromandendrene epoxide	1735	Nd	Nd	0.20 ± 0.006
	*Hydrocarbon monoterpenes*		37.19 ± 0.61	12.14 ± 0.14	2.07 ± 0.15
	*Oxygenated monoterpenes*		56.55 ± 0.30	82.89 ± 0.44	52.25 ± 2.17
	*Hydrocarbon sesquiterpenes*		3.86 ± 0.19	3.62 ± 0.22	20.74 ± 0.70
	*Oxygenated sesquiterpenes*		1.73 ± 0.14	1.12 ± 0.11	10.32 ± 1.32
	*Other*		0.60 ± 0.01	0.16 ± 0.03	14.14 ± 0.24
	***Total identified***		***99.93 ± 0.10***	***99.93 ± 0.01***	***99.52 ± 0.27***

^a^ Linear retention index on ZB-5MS column; ^b^ Expressed as the mean percentage area ± standard deviation of three repeated analyses; * Confirmed by standard; in bold the major compounds; Nd, not detected.

**Table 4:** Total phenolic and flavonoid content of extracts obtained from thyme, oregano, and basil.

Sample	Extract	TPC (mg GAE/g)	TFC (mg RE/g)
Thyme	Residual water	88.69 ± 0.45 ^a^	23.09 ± 0.08 ^c^
	Spent	66.67 ± 0.47 ^b^	28.16 ± 0.11 ^a^
	Total	65.31 ± 0.94 ^b^	24.70 ± 0.02 ^b^
Oregano	Residual water	110.35 ± 1.95 ^b^	24.95 ± 0.18 ^b^
	Spent	113.34 ± 1.71 ^ab^	34.88 ± 0.30 ^a^
	Total	115.71 ± 1.65 ^a^	25.26 ± 0.57 ^b^
Basil	Residual water	63.57 ± 0.17 ^b^	8.31 ± 0.66 ^c^
	Spent	93.66 ± 0.04 ^a^	27.59 ± 0.07 ^a^
	Total	58.85 ± 0.39 ^c^	14.03 ± 0.10 ^b^

Values are reported as mean ± SD of three parallel measurements: TPC: Total phenolic content; TFC: Total flavonoid content; GAE: Gallic acid equivalent; RE: Rutin equivalent. Different letters indicate significant differences among the extracts from each species (*p* < 0.05).

**Table 5:** Antioxidant activity of extracts obtained from thyme, oregano, and basil.

Samples	Extracts	DPPH (mg TE/g)	ABTS (mg TE/g)	CUPRAC (mg TE/g)	FRAP (mg TE/g)	MCA (mg EDTAE/g)	PBD (mmol TE/g)
Thyme	Residual water	121.00 ± 4.57 ^a^	150.09 ± 3.40 ^a^	289.24 ± 1.16 ^b^	170.15 ± 3.25 ^b^	15.04 ± 0.03 ^a^	1.77 ± 0.01 ^b^
	Spent	48.36 ± 0.06 ^b^	68.99 ± 0.63 ^b^	180.38 ± 4.66 ^c^	98.44 ± 1.55 ^c^	2.51 ± 0.54 ^b^	1.45 ± 0.04 ^c^
	Total	48.46 ± 0.09 ^b^	69.27 ± 0.09 ^b^	176.67 ± 3.03 ^c^	91.58 ± 1.75 ^d^	2.99 ± 0.14 ^b^	1.41 ± 0.02 ^c^
	Essential oil	33.44 ± 0.26 ^c^	69.58 ± 0.09 ^b^	1578.09 ± 59.67 ^a^	189.13 ± 1.52 ^a^	na	10.14 ± 0.55 ^a^
Oregano	Residual water	187.72 ± 4.15 ^c^	207.35 ± 3.84 ^c^	423.03 ± 4.31 ^c^	231.74 ± 4.15 ^c^	17.50 ± 0.13 ^a^	2.29 ± 0.03 ^c^
	Spent	266.59 ± 1.59 ^a^	285.68 ± 6.04 ^b^	575.87 ± 26.52 ^b^	319.24 ± 7.55 ^a^	2.57 ± 0.32 ^c^	2.97 ± 0.04 ^b^
	Total	246.28 ± 0.97 ^b^	347.67 ± 15.98 ^a^	538.97 ± 5.39 ^b^	291.39 ± 2.45 ^b^	6.63 ± 0.26 ^b^	2.70 ± 0.07 ^bc^
	Essential oil	37.81 ± 0.18 ^d^	69.48 ± 0.07 ^d^	1792.32 ± 33.65 ^a^	198.55 ± 4.55 ^d^	na	5.74 ± 0.34 ^a^
Basil	Residual water	48.05 ± 0.16 ^b^	69.54 ± 0.07 ^b^	188.59 ± 1.70 ^c^	97.78 ± 2.03 ^c^	10.74 ± 0.36 ^a^	1.44 ± 0.01 ^d^
	Spent	135.88 ± 1.48 ^a^	144.57 ± 2.60 ^a^	345.61 ± 5.06 ^b^	187.90 ± 4.28 ^b^	1.90 ± 0.16 ^bc^	2.14 ± 0.03 ^b^
	Total	48.49 ± 0.10 ^b^	69.36 ± 0.04 ^b^	168.24 ± 1.06 ^d^	81.63 ± 0.94 ^d^	2.12 ± 0.12 ^b^	1.53 ± 0.04 ^c^
	Essential oil	44.60 ± 0.15 ^c^	69.50 ± 0.07 ^b^	422.18 ± 23.06 ^a^	423.00 ± 4.12 ^a^	1.34 ± 0.27 ^c^	31.24 ± 1.21 ^a^

Values are reported as mean ± SD of three parallel measurements. TE: Trolox equivalent; EDTAE: EDTA equivalent; na: not active; Different letters indicate significant differences among the extracts/essential oils from each species (*p* < 0.05).

**Table 6:** Enzyme inhibitory activity of extracts obtained from thyme, oregano, and basil.

Samples	Extracts	AChE (mg GALAE/g)	BChE (mg GALAE/g)	Tyrosinase (mg KAE/g)	Amylase (mmol ACAE/g)	Glucosidase (mmol ACAE/g)
Thyme	Residual water	1.24 ± 0.04 ^c^	na	19.75 ± 0.37 ^b^	0.04 ± 0.01 ^d^	0.86 ± 0.01 ^c^
	Spent	1.71 ± 0.04 ^ab^	na	20.94 ± 0.28 ^b^	0.14 ± 0.01 ^b^	1.22 ± 0.01 ^a^
	Total	1.55 ± 0.07 ^b^	na	19.01 ± 0.27 ^b^	0.07 ± 0.01 ^c^	0.95 ± 0.01 ^b^
	Essential oil	1.80 ± 0.15 ^a^	0.52 ± 0.10	59.80 ± 3.20 ^a^	0.50 ± 0.01 ^a^	na
Oregano	Residual water	1.92 ± 0.03 ^d^	na	26.40 ± 1.27 ^c^	0.03 ± 0.01 ^d^	0.79 ± 0.01 ^c^
	Spent	2.55 ± 0.07 ^c^	na	34.89 ± 0.73 ^b^	0.19 ± 0.01 ^b^	1.27 ± 0.01 ^a^
	Total	3.27 ± 0.05 ^a^	na	34.41 ± 0.55 ^b^	0.07 ± 0.01 ^c^	0.94 ± 0.01 ^b^
	Essential oil	2.85 ± 0.17 ^b^	0.72 ± 0.05	67.01 ± 2.18 ^a^	0.60 ± 0.03 ^a^	na
Basil	Residual water	0.29 ± 0.06 ^d^	na	15.19 ± 0.99 ^c^	0.03 ± 0.01 ^d^	0.33 ± 0.01 ^c^
	Spent	1.46 ± 0.02 ^b^	na	22.06 ± 0.98 ^b^	0.24 ± 0.01 ^b^	0.97 ± 0.01 ^a^
	Total	0.59 ± 0.09 ^c^	na	16.67 ± 0.55 ^c^	0.17 ± 0.01 ^c^	0.42 ± 0.01 ^b^
	Essential oil	3.29 ± 0.13 ^a^	1.28 ± 0.03	70.00 ± 1.67 ^a^	0.66 ± 0.02 ^a^	na

Values are reported as mean ± SD of three parallel measurements. GALAE: Galanthamine equivalent; KAE: Kojic acid equivalent; Different letters indicate significant differences among the extracts/essential oils from each species (*p* < 0.05).

## Data Availability

Not applicable.

## Supplementary Materials

The following supporting information can be downloaded at: https://www.mdpi.com/article/10.3390/antiox12010210/s1, Table S1. Correlation value between chemical compounds of extracts and antioxidant abilities (R^2^). Table S2. Correlation value between chemical compounds of essential oils and antioxidant abilities (R^2^). Table S3. Correlation value between chemical compounds of extracts and enzyme inhibitory abilities (R^2^). Table S4. Correlation value between chemical compounds of essential oils and enzyme inhibitory abilities (R^2^).
