# Supplementary material for: Post-Distillation By-Products of Aromatic Plants from Lamiaceae Family as Rich Sources of Antioxidants and Enzyme Inhibitors
Source: Antioxidants (Basel). 2023 Jan 16;12(1):210. doi: 10.3390/antiox12010210 (PMC9855019; doi:10.3390/antiox12010210)
Supplement: Supplementary file 1 [file antioxidants-12-00210-s001.zip › antioxidants-2088528-Supplementary file.pdf]

Article

SUPPLEMENTARY MATERIAL for

Post-Distillation By-Products of Aromatic Plants from Lamiaceae Family as Rich Sources of Antioxidants and Enzyme Inhibitors

Simon Vlad Luca [1,*], Gokhan Zengin [2,*], Kouadio Ibrahime Sinan [2], Krystyna Skalicka-Woźniak [3] and Adriana Trifan [4]

[1] Biothermodynamics, TUM School of Life Sciences, Technical University of Munich, 85354 Freising, Germany
[2] Physiology and Biochemistry Research Laboratory, Department of Biology, Science Faculty, Selcuk University, Konya 42130, Turkey
[3] Department of Natural Products Chemistry, Medical University of Lublin, 20-093 Lublin, Poland
[4] Department of Pharmacognosy and Phytotherapy, Faculty of Pharmacy, "Grigore T. Popa" University of Medicine and Pharmacy Iasi, 700115 Iasi, Romania
* Correspondence: vlad.luca@tum.de (S.V.L.); gokhanzengin@selcuk.edu.tr (G.Z.)

Table S1. Correlation value between chemical compounds of extracts and antioxidant abilities (R^2)

**Compounds**	**DPPH**	**ABTS**	**CUPRAC**	**FRAP**	**MCA**	**PBD**
Quinic acid	-0,22	-0,18	-0,22	-0,18	0,32	-0,31
Danshensu	0,14	0,22	0,11	0,15	0,2	0,04
Hydroxybenzoic acid-O-hexoside	-0,42	-0,44	-0,4	-0,37	0,1	-0,42
Hydroxybenzoic acid	0,29	0,25	0,29	0,3	0,25	0,31
Caftaric acid	0,89	0,87	0,89	0,88	0,26	0,87
Caffeic acid-O-hexoside	-0,43	-0,45	-0,42	-0,38	0,1	-0,43
Tuberonic acid-O-hexoside	-0,47	-0,44	-0,49	-0,45	-0,01	-0,54
Roseoside	0,9	0,9	0,9	0,88	0,24	0,88
Caffeic acid	0,11	-0,03	0,13	0,11	0,01	0,2
Thymoquinol-O-hexoside	-0,28	-0,26	-0,31	-0,25	0,17	-0,35
Tuberonic acid	-0,9	-0,89	-0,9	-0,89	-0,26	-0,87
Quercetin-C-deoxyhesoide-C-hexoside	-0,47	-0,44	-0,5	-0,46	-0,02	-0,55
Fertaric acid	0,9	0,9	0,89	0,88	0,25	0,87
Luteolin-C-deoxyhexoside-C-hexoside	-0,82	-0,76	-0,83	-0,81	-0,17	-0,84
Aromadendrin-O-hexoside	-0,34	-0,32	-0,36	-0,39	-0,29	-0,28
p-Menth-1-ene-3,4-diol-O-hexoside	-0,87	-0,86	-0,87	-0,85	-0,2	-0,85
Luteolin-di-O-glucuronide	-0,47	-0,44	-0,5	-0,46	-0,02	-0,55
Quercetin-O-hexoside I	-0,42	-0,45	-0,4	-0,43	-0,25	-0,33
Quercetin-O-pentoside-O-hexoside	0,64	0,49	0,63	0,64	0,29	0,64
Taxifolin	-0,66	-0,61	-0,69	-0,67	-0,19	-0,69
Salvianolic acid H	0,4	0,41	0,37	0,4	0,24	0,3
Quercetin-O-hexoside II	0,11	-0,03	0,13	0,1	0	0,2
Gallocatechin	-0,89	-0,88	-0,88	-0,87	-0,24	-0,86
Luteolin-O-deoxyhexoside-O-hexoside	0,64	0,49	0,63	0,63	0,3	0,64
Salvianolic acid D	0,07	-0,02	0,04	0,08	0,24	0
Rosmarinic acid	-0,45	-0,34	-0,47	-0,44	-0,17	-0,51
Salvianolic acid B	-0,47	-0,44	-0,49	-0,45	-0,01	-0,54
Salvianolic acid A	-0,47	-0,44	-0,49	-0,45	-0,01	-0,54
Cichoric acid	0,9	0,88	0,9	0,88	0,25	0,88
Cymenol-O-hexoside	-0,43	-0,45	-0,41	-0,44	-0,25	-0,34
Salvianolic acid I	-0,47	-0,44	-0,49	-0,45	-0,01	-0,54
Eriodictyol	-0,42	-0,45	-0,4	-0,43	-0,26	-0,33
Salvianolic acid E	-0,47	-0,44	-0,49	-0,45	-0,01	-0,54
Salvianolic acid A isomer	0,9	0,89	0,9	0,89	0,23	0,88
Luteolin	0,07	-0,07	0,09	0,06	-0,04	0,16
Trihydroxyoctadecadienoic acid I	-0,19	-0,13	-0,17	-0,2	-0,16	-0,12
Apigenin	-0,48	-0,44	-0,5	-0,46	-0,02	-0,55
Cirsimaritin	0,43	0,39	0,45	0,41	-0,07	0,52
Trihydroxyoctadecenoic acid I	-0,43	-0,45	-0,41	-0,43	-0,25	-0,33

Ladanein	0,66	0,5	0,65	0,65	0,24	0,66
Kaempferol	-0,47	-0,44	-0,49	-0,45	-0,02	-0,54
Carvone	-0,48	-0,45	-0,5	-0,46	-0,02	-0,55
Trihydroxyoctadecadienoic acid II	-0,43	-0,45	-0,4	-0,43	-0,26	-0,33
Pebrellin	-0,42	-0,45	-0,4	-0,43	-0,27	-0,33
Cirsilineol	0,43	0,39	0,45	0,41	-0,02	0,51
Hydroperoxyoctadecadienoic acid	-0,04	-0,14	-0,02	-0,04	-0,43	0,08
Carnosol	-0,81	-0,81	-0,79	-0,81	-0,58	-0,72
Dehydrocarnosol	-0,9	-0,89	-0,89	-0,89	-0,31	-0,86
Hydroxyoctadecatrienoic acid	0,12	0,12	0,16	0,11	-0,66	0,23
4'-Hydroxy-5,5'-diisopropyl-2,2'-dimethyl-3,4-biphenylquinone	-0,61	-0,62	-0,59	-0,61	-0,71	-0,52
Hydroxyoctadecadienoic acid	0,12	0,15	0,14	0,11	-0,93	0,22
3,4,4'-Trihydroxy-5,5'-diisopropyl-2,2'-dimethylbiphenyl	-0,53	-0,56	-0,52	-0,53	-0,66	-0,43

Table S2. Correlation value between chemical compounds of essential oils and antioxidant abilities (R^2)

**Compounds**	**DPPH**	**ABTS**	**CUPRAC**	**FRAP**	**MCA**	**PBD**
Methyl 2-methylbutanoate	0,92	-0,3	-0,99	1	1	0,99
α-Phellandrene	0,72	0,06	-0,98	0,92	0,93	0,98
α-Pinene	0,89	-0,23	-1	1	1	1
Camphene	0,89	-0,21	-1	0,99	1	1
Sabinene	-0,12	-0,68	0,62	-0,47	-0,5	-0,63
β-Pinene	0,96	-0,4	-0,97	1	0,99	0,96
3-Octanone	-0,12	0,84	-0,41	0,24	0,28	0,43
β-Myrcene*	0,72	0,07	-0,97	0,92	0,93	0,98
3-Octanol	0,77	-0,01	-0,99	0,95	0,96	0,99
3-Thujene	0,43	0,41	-0,84	0,72	0,75	0,85
3-Carene	0,92	-0,3	-0,99	1	1	0,99
α-Terpinene	0,53	0,31	-0,89	0,8	0,82	0,9
p-Cymene	0,79	-0,05	-0,99	0,96	0,97	1
Limonene*	0,86	-0,17	-1	0,99	0,99	1
Eucalyptol	0,04	-0,79	0,48	-0,31	-0,35	-0,49
trans-β-Ocimene	-0,12	-0,68	0,62	-0,47	-0,5	-0,63
cis-α-Ocimene	-0,12	-0,68	0,62	-0,47	-0,5	-0,63
γ-Terpinene	0,54	0,3	-0,9	0,8	0,82	0,9
4-Pentenyl butyrate	0,92	-0,3	-0,99	1	1	0,99
cis-α-Terpineol	0,99	-0,72	-0,8	0,89	0,88	0,79
α-Terpinolene	0,5	-0,98	0,02	0,16	0,12	-0,04
p-Cymenene	0,92	-0,3	-0,99	1	1	0,99
Linalool*	0,03	-0,78	0,5	-0,33	-0,37	-0,51
trans-5-Caranol	-0,8	0,98	0,37	-0,53	-0,5	-0,35
Fenchyl alcohol	-0,12	-0,68	0,62	-0,47	-0,5	-0,63
cis-*p*-Menth-2-en-1-ol	0,69	0,11	-0,97	0,9	0,92	0,97
trans-*p*-Menth-2-en-1-ol	0,69	0,11	-0,97	0,9	0,92	0,97
Camphor	0,65	-1	-0,16	0,34	0,3	0,14
cis-Terpin hydrate	-0,12	-0,68	0,62	-0,47	-0,5	-0,63
Borneol	0,69	0,11	-0,96	0,9	0,92	0,97
Terpinen-4-ol	0,58	0,25	-0,92	0,83	0,85	0,93
p-Cymen-8-ol	0,85	-0,15	-1	0,98	0,99	1
Estragole	-0,12	-0,68	0,62	-0,47	-0,5	-0,63
trans-α-Terpineol	0,57	0,27	-0,91	0,82	0,84	0,92
Dihydrocarvone	-0,8	0,98	0,37	-0,53	-0,5	-0,35
Octyl acetate	-0,12	-0,68	0,62	-0,47	-0,5	-0,63
cis-Geraniol	-0,12	-0,68	0,62	-0,47	-0,5	-0,63

Thymol methyl ether	0,92	-0,3	-0,99	1	1	0,99
Isothymol methyl ether	0,54	0,3	-0,9	0,8	0,82	0,9
d-Darvone	-0,69	-0,11	0,97	-0,9	-0,92	-0,97
trans-Geraniol	0,42	-0,97	0,11	0,07	0,04	-0,12
m-Cymene	0,92	-0,3	-0,99	1	1	0,99
Thymol isomer	0,87	-0,18	-1	0,99	0,99	1
Bornyl acetate	-0,12	-0,68	0,62	-0,47	-0,5	-0,63
Thymol	0,81	-0,08	-1	0,97	0,97	1
Carvacrol	-0,76	0,99	0,31	-0,48	-0,45	-0,3
(Z)-Methyl cinnamate	-0,12	-0,68	0,62	-0,47	-0,5	-0,63
Isoeugenol	-0,8	0,98	0,37	-0,53	-0,5	-0,35
2-Hydroxycineole acetate	-0,12	-0,68	0,62	-0,47	-0,5	-0,63
Eugenol	-0,1	-0,7	0,6	-0,45	-0,48	-0,61
Isobornyl propionate	0,92	-0,3	-0,99	1	1	0,99
Copaene	0,4	-0,96	0,14	0,05	0,01	-0,15
α-Farnesene	0,92	-0,3	-0,99	1	1	0,99
(*E*)-Methyl cinnamate	-0,12	-0,68	0,62	-0,47	-0,5	-0,63
β-Elemene	-0,12	-0,68	0,62	-0,47	-0,5	-0,63
Methyl eugenol	-0,12	-0,68	0,62	-0,47	-0,5	-0,63
(*E*)-α-Bergamotene	-0,12	-0,68	0,62	-0,47	-0,5	-0,63
Caryophyllene*	0,57	0,26	-0,91	0,83	0,85	0,92
(*Z*)-α-Bergamotene	-0,12	-0,68	0,62	-0,47	-0,5	-0,63
4-*t*-Butyl-pyrocatechol	0,82	-0,1	-1	0,97	0,98	1
γ-Elemene	-0,89	0,22	1	-0,99	-1	-1
β-Farnesene	-0,12	-0,68	0,62	-0,47	-0,5	-0,63
Cedrene	-0,12	-0,68	0,62	-0,47	-0,5	-0,63
Humulene*	-0,28	-0,55	0,74	-0,6	-0,63	-0,75
Nerol acetate	0,92	-0,3	-0,99	1	1	0,99
β-Cubenene	-0,12	-0,68	0,62	-0,47	-0,5	-0,63
α-Himalachene	-0,12	-0,68	0,62	-0,47	-0,5	-0,63
α-Huaiene	0,74	-0,99	-0,29	0,46	0,43	0,28
Germacrene D	-0,12	-0,68	0,62	-0,47	-0,5	-0,63
β-Selinene	0,08	-0,81	0,45	-0,28	-0,32	-0,47
α-Selinene	0,08	-0,81	0,45	-0,28	-0,32	-0,47
β-Bisabolene	-1	0,57	0,9	-0,96	-0,95	-0,89
γ-Cadinene	-0,06	-0,73	0,57	-0,41	-0,44	-0,58
β-Cadinene	0,71	0,08	-0,97	0,91	0,93	0,98
δ-Cadinene	0,02	-0,78	0,5	-0,34	-0,37	-0,52
α-Bisabolene	-0,12	-0,68	0,62	-0,47	-0,5	-0,63

(*E*)-Farnesene epoxide	-0,12	-0,68	0,62	-0,47	-0,5	-0,63
Nerolidol	-0,12	-0,68	0,62	-0,47	-0,5	-0,63
Globulol	-0,12	-0,68	0,62	-0,47	-0,5	-0,63
Spathulenol	-0,16	-0,65	0,65	-0,5	-0,53	-0,66
β-Caryophyllene oxide*	0,23	0,59	-0,7	0,56	0,59	0,71
Aromadendrene oxide	-0,12	-0,68	0,62	-0,47	-0,5	-0,63
cis-(*Z*)-α-Bisabolene epoxide	-0,12	-0,68	0,62	-0,47	-0,5	-0,63
trans-(*E*)-α-Bisabolene epoxide	-0,8	0,98	0,37	-0,53	-0,5	-0,35
Cubenol	-0,01	-0,76	0,53	-0,36	-0,4	-0,54
γ-Eudesmol	0,92	-0,3	-0,99	1	1	0,99
trans-(*Z*)-α-Bisabolene epoxide	-0,12	-0,68	0,62	-0,47	-0,5	-0,63
α-Cadinol	-0,08	-0,71	0,58	-0,43	-0,46	-0,6
β-Eudesmol	-0,03	-0,75	0,54	-0,38	-0,42	-0,56
allo-Aromadendrene epoxide	0,34	-0,94	0,2	-0,02	-0,05	-0,21
α-Bisabolol*	-0,12	-0,68	0,62	-0,47	-0,5	-0,63
Ledene alcohol	-0,12	-0,68	0,62	-0,47	-0,5	-0,63
Ledene oxide	-0,12	-0,68	0,62	-0,47	-0,5	-0,63
Isoaromandendrene epoxide	-0,12	-0,68	0,62	-0,47	-0,5	-0,63

Table S3. Correlation value between chemical compounds of extracts and enzyme inhibitory abilities (R^2)

Compounds	AChE	Tyrosinase	Amylase	Glucosidase
Quinic acid	0,01	-0,21	-0,45	0,22
Danshensu	0,46	0,26	-0,35	0,57
Hydroxybenzoic acid-O-hexoside	-0,48	-0,5	0,05	-0,09
Hydroxybenzoic acid	-0,02	0,17	0,21	-0,04
Caftaric acid	0,77	0,89	-0,1	0,33
Caffeic acid-O-hexoside	-0,46	-0,5	0,04	-0,04
Tuberonic acid-O-hexoside	-0,1	-0,35	-0,27	0,35
Roseoside	0,8	0,91	-0,12	0,33
Caffeic acid	-0,35	-0,05	0,38	-0,4
Thymoquinol-O-hexoside	-0,09	-0,23	-0,15	0,32
Tuberonic acid	-0,8	-0,9	0,12	-0,35
Quercetin-C-deoxyhesoide-C-hexoside	-0,1	-0,35	-0,26	0,35
Fertaric acid	0,8	0,9	-0,13	0,32
Luteolin-C-deoxyhexoside-C-hexoside	-0,52	-0,74	-0,09	-0,08
Aromadendrin-O-hexoside	-0,42	-0,34	0,29	-0,52
p-Menth-1-ene-3,4-diol-O-hexoside	-0,78	-0,88	0,11	-0,31
Luteolin-di-O-glucuronide	-0,1	-0,35	-0,27	0,35
Quercetin-O-hexoside I	-0,68	-0,55	0,39	-0,67
Quercetin-O-pentoside-O-hexoside	0,38	0,58	0,03	0,31
Taxifolin	-0,36	-0,55	-0,08	0,01
Salvianolic acid H	0,66	0,52	-0,37	0,68
Quercetin-O-hexoside II	-0,35	-0,05	0,39	-0,41
Gallocatechin	-0,81	-0,91	0,11	-0,39
Luteolin-O-deoxyhexoside-O-hexoside	0,38	0,58	0,02	0,3
Salvianolic acid D	0,22	0,14	-0,23	0,59
Rosmarinic acid	-0,01	-0,28	-0,28	0,25
Salvianolic acid B	-0,1	-0,35	-0,27	0,35
Salvianolic acid A	-0,1	-0,35	-0,27	0,35
Cichoric acid	0,78	0,9	-0,1	0,33
Cymenol-O-hexoside	-0,69	-0,55	0,38	-0,69
Salvianolic acid I	-0,1	-0,35	-0,27	0,35
Eriodictyol	-0,68	-0,54	0,41	-0,66
Salvianolic acid E	-0,1	-0,35	-0,27	0,35
Salvianolic acid A isomer	0,79	0,91	-0,09	0,34
Luteolin	-0,38	-0,1	0,43	-0,43
Trihydroxyoctadecadienoic acid I	-0,45	-0,31	0,28	-0,64

Apigenin	-0,1	-0,35	-0,26	0,36
Cirsimaritin	0,05	0,31	0,36	-0,35
Trihydroxyoctadecenoic acid I	-0,69	-0,55	0,38	-0,68
Ladanein	0,4	0,61	0,07	0,33
Kaempferol	-0,1	-0,35	-0,25	0,36
Carvone	-0,1	-0,35	-0,26	0,35
Trihydroxyoctadecadienoic acid II	-0,68	-0,55	0,39	-0,67
Pebrellin	-0,68	-0,54	0,41	-0,66
Cirsilineol	0,05	0,31	0,32	-0,36
Hydroperoxyoctadecadienoic acid	-0,41	-0,15	0,65	-0,33
Carnosol	-0,63	-0,72	0,38	-0,3
Dehydrocarnosol	-0,78	-0,89	0,17	-0,33
Hydroxyoctadecatrienoic acid	0,27	0,29	0,5	0,11
4'-Hydroxy-5,5'-diisopropyl-2,2'-dimethyl-3,4-biphenylquinone	-0,31	-0,47	0,57	0,08
Hydroxyoctadecadienoic acid	0,38	0,31	0,74	0,43
3,4,4'-Trihydroxy-5,5'-diisopropyl-2,2'-dimethylbiphenyl	-0,35	-0,45	0,7	0,04

Table S4. Correlation value between chemical compounds of essential oils and enzyme inhibitory abilities (R^2)

Compounds	AChE	BChE	Tyrosinase	Amylase
Methyl 2-methylbutanoate	0,73	0,97	0,73	0,76
α-Phellandrene	0,44	0,81	0,43	0,48
α-Pinene	0,68	0,95	0,68	0,72
Camphene	0,67	0,94	0,67	0,7
Sabinene	0,23	-0,26	0,23	0,18
β-Pinene	0,8	0,99	0,8	0,83
3-Octanone	-0,45	0,02	-0,46	-0,41
β-Myrcene*	0,43	0,81	0,43	0,48
3-Octanol	0,51	0,85	0,5	0,55
3-Thujene	0,09	0,55	0,09	0,14
3-Carene	0,73	0,97	0,73	0,76
α-Terpinene	0,21	0,64	0,2	0,25
p-Cymene	0,54	0,87	0,53	0,58
Limonene*	0,64	0,93	0,63	0,67
Eucalyptol	0,39	-0,09	0,39	0,34
trans-β-Ocimene	0,23	-0,26	0,23	0,18
cis-α-Ocimene	0,23	-0,26	0,23	0,18
γ-Terpinene	0,21	0,65	0,2	0,26
4-Pentenyl butyrate	0,73	0,97	0,73	0,76
cis-α-Terpineol	0,97	0,97	0,97	0,98
α-Terpinolene	0,77	0,37	0,77	0,74
p-Cymenene	0,73	0,97	0,73	0,76
Linalool*	0,37	-0,11	0,37	0,32
trans-5-Caranol	-0,96	-0,71	-0,96	-0,94
Fenchyl alcohol	0,23	-0,26	0,23	0,18
cis-*p*-Menth-2-en-1-ol	0,4	0,78	0,39	0,44
trans-*p*-Menth-2-en-1-ol	0,4	0,78	0,39	0,44
Camphor	0,87	0,54	0,87	0,85
cis-Terpin hydrate	0,23	-0,26	0,23	0,18
Borneol	0,39	0,78	0,39	0,44
Terpinen-4-ol	0,26	0,69	0,26	0,31
p-Cymen-8-ol	0,62	0,92	0,61	0,65
Estragole	0,23	-0,26	0,23	0,18
trans-α-Terpineol	0,25	0,68	0,24	0,29
Dihydrocarvone	-0,96	-0,71	-0,96	-0,94
Octyl acetate	0,23	-0,26	0,23	0,18
cis-Geraniol	0,23	-0,26	0,23	0,18

Thymol methyl ether	0,73	0,97	0,73	0,76
Isothymol methyl ether	0,22	0,65	0,21	0,26
d-Darvone	-0,4	-0,78	-0,39	-0,44
trans-Geraniol	0,71	0,3	0,72	0,68
m-Cymene	0,73	0,97	0,73	0,76
Thymol isomer	0,65	0,93	0,64	0,68
Bornyl acetate	0,23	-0,26	0,23	0,18
Thymol	0,56	0,88	0,55	0,6
Carvacrol	-0,94	-0,66	-0,94	-0,92
(Z)-Methyl cinnamate	0,23	-0,26	0,23	0,18
Isoeugenol	-0,96	-0,71	-0,96	-0,94
2-Hydroxycineole acetate	0,23	-0,26	0,23	0,18
Eugenol	0,25	-0,24	0,26	0,2
Isobornyl propionate	0,73	0,97	0,73	0,76
Copaene	0,69	0,27	0,69	0,66
α-Farnesene	0,73	0,97	0,73	0,76
(*E*)-Methyl cinnamate	0,23	-0,26	0,23	0,18
β-Elemene	0,23	-0,26	0,23	0,18
Methyl eugenol	0,23	-0,26	0,23	0,18
(*E*)-α-Bergamotene	0,23	-0,26	0,23	0,18
Caryophyllene*	0,25	0,68	0,25	0,3
(*Z*)-α-Bergamotene	0,23	-0,26	0,24	0,18
4-*t*-Butyl-pyrocatechol	0,58	0,89	0,57	0,62
γ-Elemene	-0,68	-0,94	-0,67	-0,71
β-Farnesene	0,23	-0,26	0,23	0,18
Cedrene	0,23	-0,26	0,23	0,18
Humulene*	0,07	-0,41	0,07	0,02
Nerol acetate	0,73	0,97	0,73	0,76
β-Cubenene	0,23	-0,26	0,23	0,18
α-Himalachene	0,23	-0,26	0,23	0,18
α-Huaiene	0,93	0,65	0,93	0,91
Germacrene D	0,23	-0,26	0,23	0,18
β-Selinene	0,42	-0,06	0,42	0,37
α-Selinene	0,42	-0,06	0,42	0,37
β-Bisabolene	-0,9	-1	-0,9	-0,92
γ-Cadinene	0,29	-0,2	0,29	0,24
β-Cadinene	0,42	0,8	0,42	0,47
δ-Cadinene	0,36	-0,12	0,37	0,32
α-Bisabolene	0,23	-0,26	0,23	0,18
(*E*)-Farnesene epoxide	0,23	-0,26	0,23	0,18

Nerolidol	0,23	-0,26	0,23	0,18
Globulol	0,23	-0,26	0,23	0,18
Spathulenol	0,19	-0,3	0,19	0,14
β-Caryophyllene oxide*	-0,12	0,37	-0,12	-0,07
Aromadendrene oxide	0,23	-0,26	0,23	0,18
cis-(*Z*)-α-Bisabolene epoxide	0,23	-0,26	0,23	0,18
trans-(*E*)-α-Bisabolene epoxide	-0,96	-0,71	-0,96	-0,94
Cubenol	0,34	-0,15	0,34	0,29
γ-Eudesmol	0,73	0,97	0,73	0,76
trans-(*Z*)-α-Bisabolene epoxide	0,23	-0,26	0,23	0,18
α-Cadinol	0,27	-0,22	0,28	0,23
β-Eudesmol	0,32	-0,17	0,32	0,27
allo-Aromadendrene epoxide	0,64	0,21	0,65	0,61
α-Bisabolol*	0,23	-0,26	0,23	0,18
Ledene alcohol	0,23	-0,26	0,23	0,18
Ledene oxide	0,23	-0,26	0,23	0,18
Isoaromandendrene epoxide	0,23	-0,26	0,23	0,18
